# Cimicifuga, Corrigendum

**Published:** 1887-05

**Authors:** Wm. Jefferson Guernsey

**Affiliations:** Philadelphia


### CIMICIFUGA—CORRIGENDUM.

Wm. Jefferson Guernsey, M. D., Philadelphia.

In the August (1886) number of the St. Louis *Periscope*, p. 290, appeared an article by myself, entitled "Homœopathic Prophylactics," a little paper prepared for the "International Hahnemannian Association." At the bottom of page 292, in said article, will be found a reference to the use of Cimicifuga as a means of rendering labor short and easy. I am glad that I therein intimated that I had not experimented with it myself, and regret that I did not wait until I had thus proven its value (or cussedness, as I now believe) before rushing it into print. It is not a new suggestion, and doubtless every practitioner has heard it recommended for that purpose, but my California friend laid such stress upon its value that I did not hesitate to give it the prominence it seemed to demand. Since that date I have had some sad experiences with it, and it is with a sole desire of warning others *not* to use it that I now write.

I determined to prescribe it in ten cases and note the result. I administered it as advised, using the one thousandth potency (Tafel), which I knew to be good, until the thought occurred to me that, as my friend was a low dilutionist, I would not follow in his successes unless I employed the potency he had doubtless used, and then gave to two cases the 3x Bœricke.

Case I was confined before I could reach the house, although found without delay. I was in great glee until I learned that this had been her *usual programme*, a fact I had neglected to ascertain. Case II said she thought she had "rather a better time of it," but if so I am thankful it did not fall to my lot to attend her, as she was on this occasion sick twelve hours after I arrived (having been in agony for hours before), and from which condition I finally relieved her by use of forceps, a procedure I much dislike. Case III said she was longer and suffered more than ever before. Case IV had an exceedingly long and difficult labor. Case V said she thought her labor was rather easier, but as I had attended her only eighteen months before I thought that she had appeared to suffer more than at the last labor, the suffering of which she may have partly forgotten "in joy that a man was born." Cases VII, VIII, and X deserve no special mention, save that they were not favorably affected by the remedy. It is with cases VI and IX that I have most to say, and it is an interesting fact that those two, the most difficult cases, should have received different potencies, one the 3x and the other the M. *Before labor:* Severe labor-like pains, preventing sleep and lasting at intervals for several weeks—both cases. (Some of the others had similar pains, though less severe.) *During labor:* When labor finally set in, which was much over the calculated time, the pains were terribly severe and ineffectual; she would cry out in perfect frenzy, because her child could not be born, and upon examination but little progress could be detected. *After labor:* Severe after-pains; sleeplessness; chilliness on motion; fever; cold sweat; cessation of lochia; copious, frequent, watery, frothy stools, accompanied by pain and followed by prostration; craving for beer; marked despondency (thinks she will die); headache; viscid mucus in mouth, as well as throat, which is very offensive to patient and exceedingly difficult to detach, causing nausea; urine dark, scant, and brownish; delirium one night (one case only); cough; sensitiveness to noise; one patient had a clock stopped that had hung inoffensively at the opposite end of her bed-room for years; complete anorexia; abdominal pain (indescribable), with soreness there. The above is a picture of both cases, though one was much more severe; the symptoms were grave and were difficult to remove—*Verat.* being of more service than any other drug in relieving the diarrhœa and cold sweat and generally low condition and restoring the lochia. While attending the first case I wrote to Dr. C. Carleton Smith stating symptoms, having all the time in my mind the fear that I had caused the trouble, but neglecting to mention to him the fact of using the drug. His reply was, "Use Cimicif.; it has all the symptoms." Imagine my wrath. No antidote could be found in the books, so I concluded to try a different potency and gave the 2c in water with a relief of some symptoms.

Whether the Western climate has aught to do with my friend's successes in this matter is a question—at all events, it is not the drug for Philadelphians to trifle with.

The only prophylactic that I *know* to be of real value is the indicated homœopathic medicine. Few pregnant females are exempt from some aches and pains or abnormal mental conditions, and these symptoms furnish the sole indication for the remedy that will lighter labor. *This* others have proven. *This* I have again and again tested, and upon it hang my sole hopes of ameliorating that which can never be cured, O woman! for "in *sorrow* thou shalt bring forth children."

